# Aortoiliac Occlusive Disease: When the Development of Arterial Collateral Network Takes Over

**DOI:** 10.1055/s-0040-1714078

**Published:** 2020-12-11

**Authors:** Efstratios Georgakarakos, Ioannis Katsaros, Aliki Fiska

**Affiliations:** 1 Department of Vascular Surgery, “Democritus” University of Thrace, University Hospital of Alexandroupolis, Alexandroupolis, Greece; 2 Department of Anatomy, Medical School of Alexandroupolis, Democritus University of Thrace, Alexandroupolis, Greece

**Keywords:** aortoiliac occlusive disease, Leriche's syndrome, collateral circulation, anatomy

## Abstract

This report describes the collateral pathways that restore arterial circulation in cases of aortoiliac occlusive disease and discusses the clinical and surgical importance of these systemic-systemic, visceral-systemic, and visceral-visceral anastomoses.

An 85-year old male presented with severe intermittent claudication bilaterally. Computed tomography angiography showed complete occlusion of the common and external iliac artery on the left and occlusion of the external iliac artery on the right ( Fig. 1 ). In such cases, the perfusion of limbs is attributed to an extensive collateral network between the systemic-systemic, visceral-systemic, and visceral-visceral pathways. 1 2 The celiac, superior, and inferior mesenteric arteries form collateral pathways (white arrowhead) that perfuse the hypogastric arteries through the superior rectal and the middle sacral arteries (white arrow) or the branches (yellow arrowhead) of the common and deep femoral arteries via the pelvis transverse collateral pathway (obturator, internal pudendal, and symphyseal arteries). The adequacy of this mechanism depends on the enlargement of preexisting vessels, although individuals differ in their capacity to develop collateral vessels. 3 Age, diabetes, and hypertension suppress collateral development. Oxidative stress and endothelial dysfunction influence the capacity for collateral growth, while increased wall shear stress and circumferential wall tension due to dilation can lead to the luminal expansion and medial thickening observed in collateral arteries. 3 These factors can be modified by a supervised training/walking program and pharmaceutical agents, such as statins and angiotensin-converting enzyme-inhibitors.

**Fig. 1:**
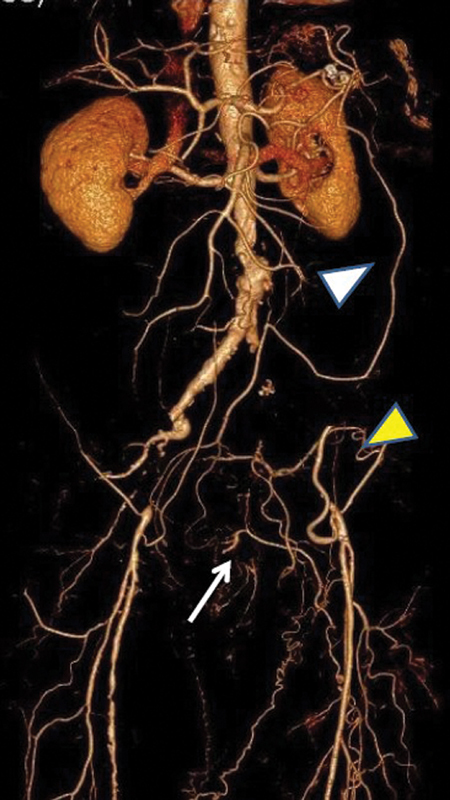
Computed tomography angiography of the patient with complete occlusion of the left common and external iliac artery and occlusion of the right external iliac artery. A complex network of systemic and visceral anastomotic vessels restores the flow to the lower limbs (for details see in the text).
